# Amphetamine-Induced OCD-Related Repetitive Behaviors Are Potentiated in *Slc1a1-OE* Mice

**DOI:** 10.1523/ENEURO.0409-24.2024

**Published:** 2024-10-16

**Authors:** Esther Y. Choi

**Affiliations:** Department of Neuroscience, Western University, London, Ontario N6A 3K7, Canada

Obsessive–compulsive disorder (OCD) is a neuropsychiatric disorder characterized by obsessions and compulsions. Obsessions are intrusive thoughts or urges, and compulsions are repetitive behaviors that a person often performs to alleviate the anxiety related to these obsessions. The obsessions and compulsions associated with OCD can be debilitating, affecting the daily lives and overall quality of life for those with OCD (Pauls et al., 2014). OCD has a lifetime prevalence of ∼2–3% and although there are treatments for those diagnosed with OCD, unfortunately up to 50% of these patients continue to display symptoms (Dougherty et al., 2004). Better, more targeted treatment options are needed, and understanding the neurobiology of OCD is important in improving the treatment options for those affected by this disorder.

Previous studies of OCD patients using neuroimaging techniques have identified increased activity of cortical and striatal regions (Pauls et al., 2014). Additionally, animal studies have also implicated the dysfunction of specific glutamate signaling circuits in OCD-relevant behaviors (Welch et al., 2007). With respect to genetic risk, the chromosome region 9p24, containing the gene *SLC1A1*, has been implicated in the development of OCD. Furthermore, the rs301430 polymorphism has been replicated in several OCD association studies, and this polymorphism increases *SLC1A1* expression in humans, including in the brain tissue. *SLC1A1* is a gene that encodes the protein EAAT3, which is a neuronal glutamate transporter. Thus, the increase in *SLC1A1* expression observed with this polymorphism also leads to increased expression and activity of EAAT3 (Veenstra-VanderWeele et al., 2012). These studies suggest that there may be an increased risk of OCD and OCD-relevant behaviors in model systems with elevated *SLC1A1*/EAAT3 levels. Additionally, animal studies have displayed a relationship between *Slc1a1*/EAAT3 and abnormal repetitive behaviors such as a significant increase in amphetamine-induced repetitive behaviors in mice with increased expression of *Slc1a1* (Escobar et al., 2021).

In the recent eNeuro publication by Kopelman et al. (2024), the authors used a novel *Slc1a1*-overexpressing (OE) mouse model with elevated EAAT3 expression in forebrain regions to investigate the relationship between EAAT3 and amphetamine-induced repetitive behavior and striatal activation. The authors created this novel mouse model by crossing *Slc1a1*-tetO-STOP mice with *Pgk1*-flpo mice first, then crossing the offspring of these mice with *CaMKII*-tetracycline transactivator (tTA) mice which allowed for *Slc1a1* to be selectively overexpressed in forebrain neurons in a doxycycline-dependent manner. The authors then confirmed that the expression of EAAT3 is selective and reversible in the forebrain of *Slc1a1*-OE mice.

Baseline anxiety testing revealed no significant differences between tTA-control and *Slc1a1*-OE mice in open-field and light/dark behavioral paradigms. Additionally, the authors tested repetitive behaviors by observing OCD-related grooming behaviors in the mice but did not see any significant difference in either the baseline or drug-induced (D1-agonist SKF-38393) grooming behaviors exhibited. However, when mice were injected with low-dose amphetamine (3.0 mg/kg), compared with the tTA-control mice, the *Slc1a1*-OE mice showed significantly increased locomotor activity. The authors then injected the mice with high-dose amphetamine (8.0 mg/kg) and observed more stereotypy, characterized as stationary head bobbing, sniffing, shuffling, or licking behavior lasting at least 1 s, compared with the control group. These results were consistent regardless of whether EAAT3 was overexpressed throughout the lifetime or specifically in adulthood.

Previous research has implicated the involvement of striatal regions in OCD-relevant behaviors (Welch et al., 2007). In order to explore what neural activity may be associated with the specific behavior observed with administration of amphetamine in *Slc1a1*-OE mice, the authors measured c-Fos, a marker for neuronal activity in the striatum, in mice injected with high-dose amphetamine. A significant increase in c-Fos expression in the ventral striatum was observed.

In addition to the behavioral and immunohistochemistry assays, Kopelman et al. (2024) ran a novel machine learning program to further analyze the behavior in *Slc1a1-*OE mice in an unbiased manner. This algorithm, known as behavioral segmentation of open field in DeepLabCut, is an unsupervised learning algorithm that can classify behaviors in an unbiased fashion. Their results using this algorithm uncovered six distinct clusters of behavior, one of which correspond to the stereotypy behavior, and importantly, they showed results that aligned with the previously hand-scored results. They also performed further c-Fos analysis on this cohort and found that this machine-classified behavior correlated with increased c-Fos in D1 neurons in the ventromedial striatum in mice receiving amphetamine.

However, as it is well discussed in the paper, there are some limitations of this study, such as the lack of in vivo temporal dynamics of D1 and D2 receptor expression. Studies with in vivo recording methods would be important to better understand the relationship between the observed behaviors and these neurons. In addition, the association between polymorphisms in *Slc1a1* and OCD has not been replicated in larger genome-wide association studies. Nonetheless, this innovative *Slc1a1*-OE mouse model, in combination with the behavioral assays and novel unbiased machine learning analysis, has provided a method to examine the repetitive, OCD-related behaviors triggered by administration of amphetamine.

Given the substantial effects that OCD has on individuals globally, this paper offers valuable insights into the involvement of *Slc1a1* in OCD-related behaviors. As explained by the authors in the paper, while the atypical repetitive behaviors induced by amphetamine may not only represent OCD symptoms and could also be related to other conditions such as tic disorders, there may be neural mechanism overlaps between OCD and these amphetamine-induced behaviors. Therefore, this study has opened the door for forthcoming research aimed at further examining the potential correlation between this gene and OCD. As a result, this may potentially contribute to the development or advancement of novel treatments for OCD, which may offer benefits to many individuals worldwide.
